# Supplementary material for: Formation of nitrogen-containing gas phase products from the heterogeneous (photo)reaction of NO2 with gallic acid
Source: Commun Chem. 2023 Sep 16;6:198. doi: 10.1038/s42004-023-01003-3 (PMC10505156; doi:10.1038/s42004-023-01003-3)
Supplement: Supplementary file 2 — Supplementary Information [file 42004_2023_1003_MOESM2_ESM.pdf]

Supplementary Information

Formation of nitrogen-containing gas phase products from the heterogeneous (photo)reaction of NO_2 with gallic acid

Pan Li[1,2,3,4], Rachel Gemayel[5], Xue Li[6,7,8], Jiangping Liu[9], Mingjin Tang[1,2,3,4], Xinming Wang[1,2,3,4], Yan Yang[10,11,12]*, Hind A. Al-Abadleh[13]*, Sasho Gligorovski[1,2,3,]*

[1]State Key Laboratory of Organic Geochemistry and Guangdong Provincial Key Laboratory of Environmental Protection and Resources Utilization, Guangzhou Institute of Geochemistry, Chinese Academy of Sciences, Guangzhou 510 640, China
[2]Guangdong-Hong Kong-Macao Joint Laboratory for Environmental Pollution and Control, Guangzhou Institute of Geochemistry, Chinese Academy of Science, Guangzhou 510640, China
[3]Chinese Academy of Science, Center for Excellence in Deep Earth Science, Guangzhou, 510640
[4]University of Chinese Academy of Sciences, Beijing, China
[5]Institut National de l'Environnement industriel et des RISques (INERIS), Parc technologique Alata BP2, 60550 Verneuil en Halatte, France
[6]Institute of Mass Spectrometry and Atmospheric Environment, Jinan University, Guangzhou 510632, China
[7]Guangdong Provincial Engineering Research Center for On-line Source Apportionment System of Air Pollution, Guangzhou, 510632, China
[8]Guangdong-Hongkong-Macau Joint Laboratory of Collaborative Innovation for Environmental Quality, Guangzhou 510632, China
[9]Faculty of Environmental Science and Engineering, Kunming University of Science and Technology, Kunming, 650500, China
[10]School of Environmental Science and Engineering, Guangdong University of Technology, Guangzhou, 510006, Guangdong, China
[11]Jieyang Branch of Chemistry and Chemical Engineering Guangdong Laboratory(Rongjiang Laboratory), Jieyang 515200, China
[12]Synergy Innovation Institute of GDUT, Shantou, 515041, Guangdong, China
[13]Department of Chemistry and Biochemistry, Wilfrid Laurier University, Waterloo, ON N2L 3C5, Canada

*Corresponding authors:
Sasho Gligorovski
gligorovski@gig.ac.cn
Hind A. Al-Abadleh
halabadleh@wlu.ca
Yan Yang
yangyan1209@gdut.edu.cn

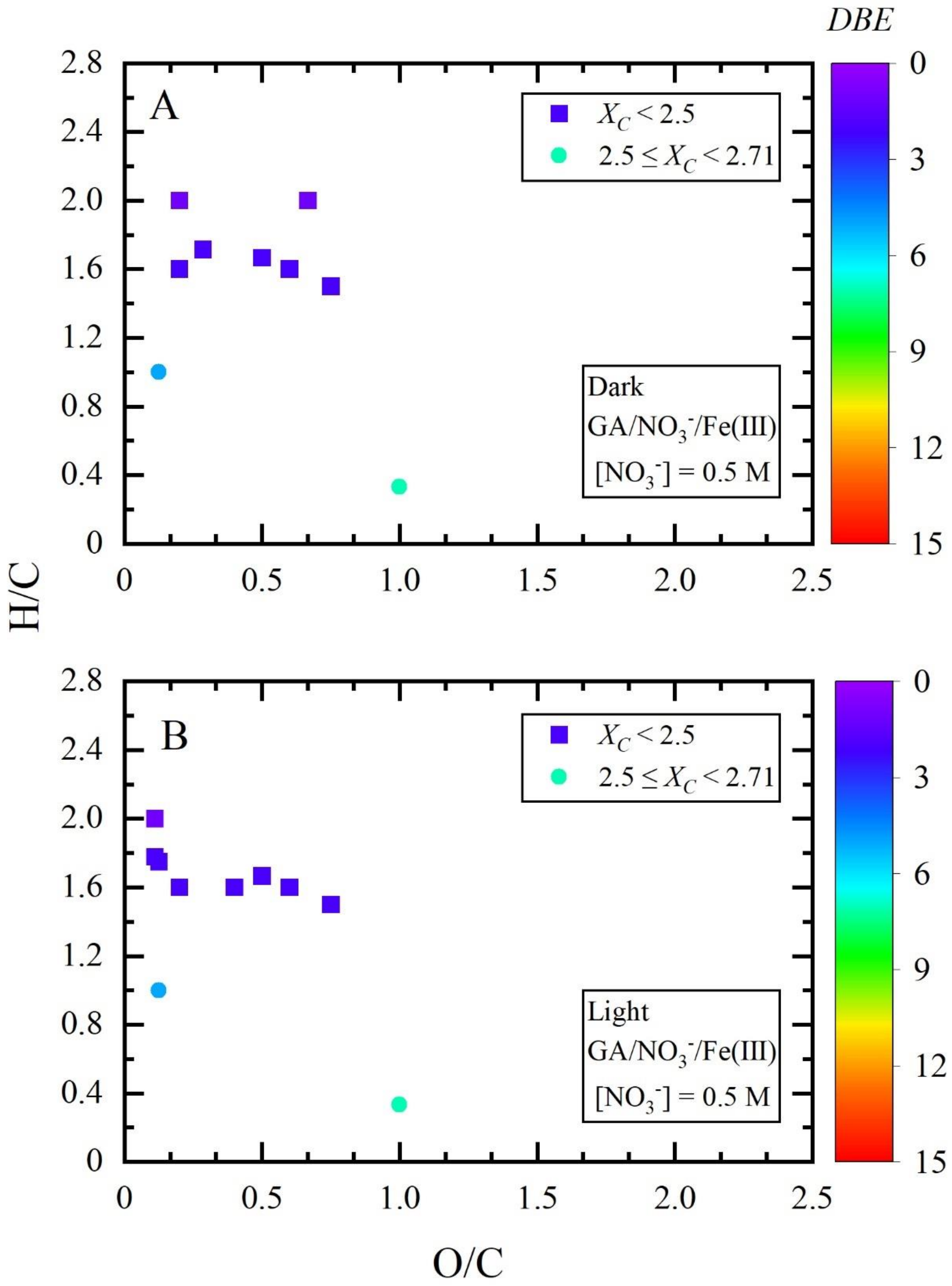

Figure S1: Van Krevelen plot showing the correlation between H/C ratio and O/C ratio obtained for the organic compounds detected during the heterogeneous reaction of NO_2 with GA/NO_3^-/Fe(III) ([NO_3^-] = 0.5 mol L^{-1}). (A) GA/NO_3^-/Fe(III) ([NO_3^-] = 0.5 mol L^{-1}) in dark, and (B) GA/NO_3^-/Fe(III) ([NO_3^-] = 0.5 mol L^{-1}) under irradiation.

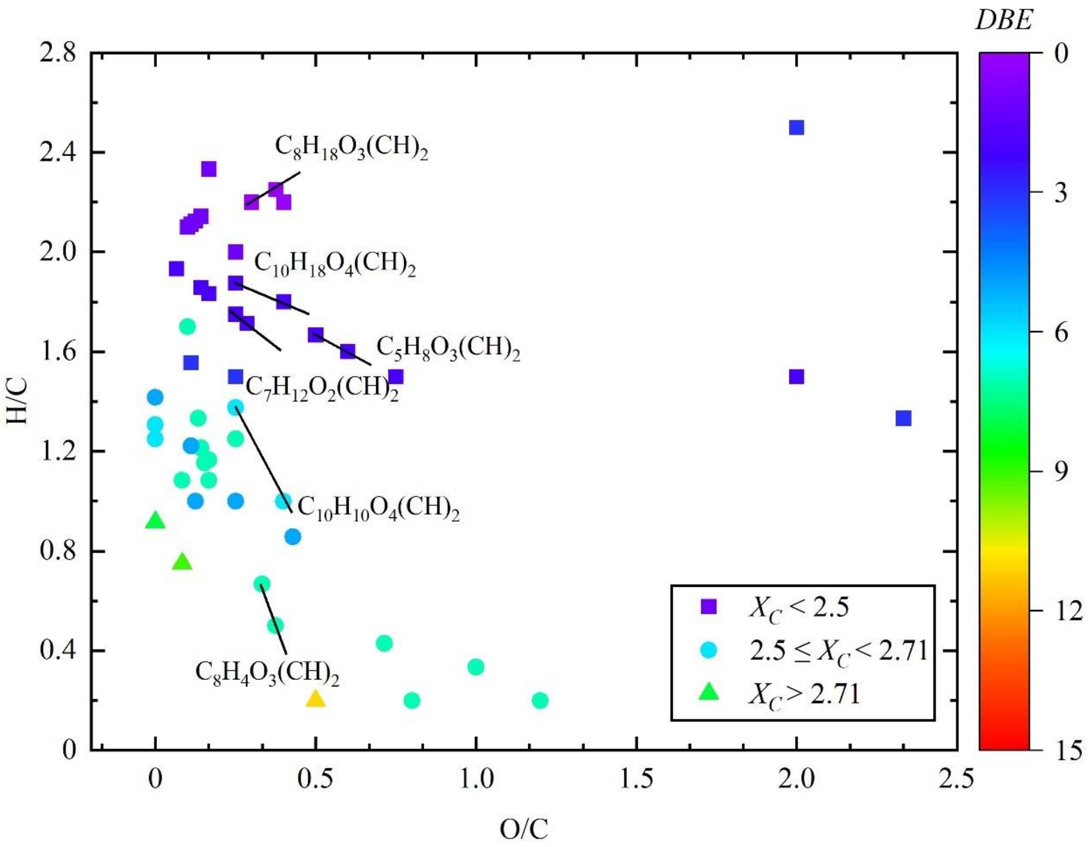

Figure S2: Van Krevelen plot showing the correlation between H/C ratio with O/C ratio obtained for the gas phase compounds detected in heterogeneous reaction of NO_2 with GA/Fe(III)/NO_3^- ([NO_3^-] = 0.05 mol L^{-1}) in dark.

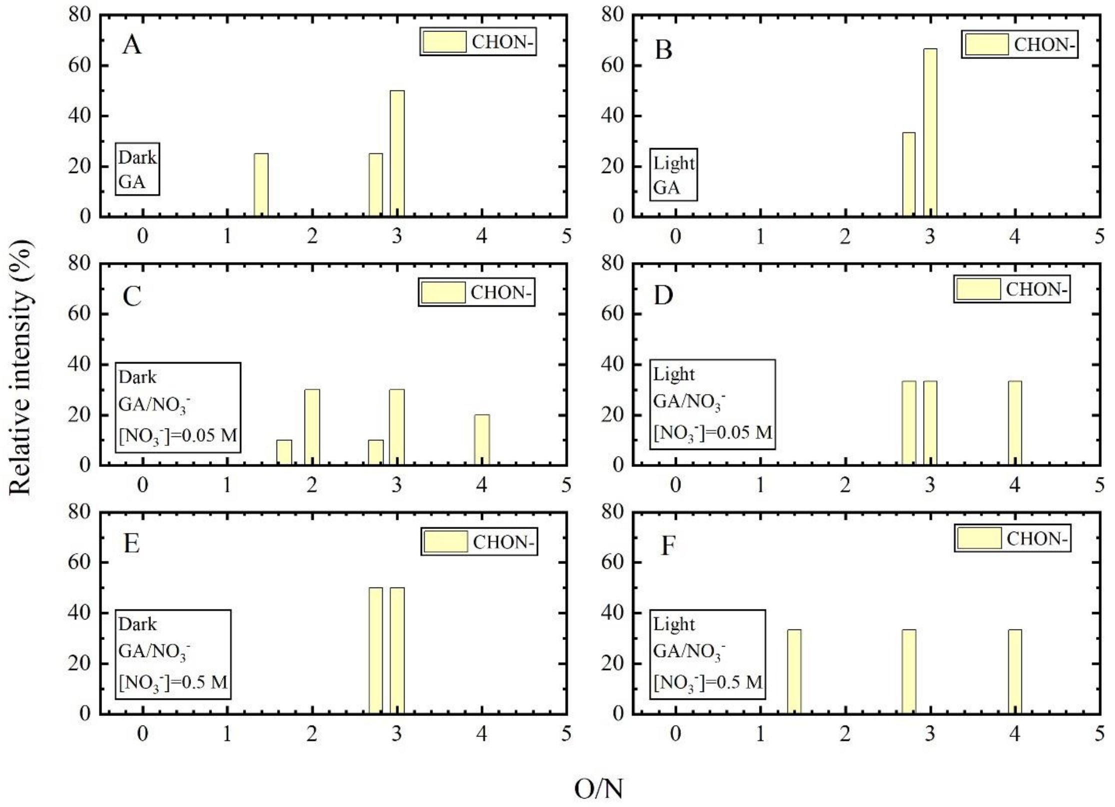

Figure S3: N-containing organic compounds are divided into subgroups related to the O/N ratio in their chemical composition. The y-axis represents the contribution of each subgroup to the total intensity of CHON compounds in ESI^- ion mode detected during the heterogeneous reaction of NO_2 with GA (A) in dark, and (B) under irradiation (C) GA/NO_3^- ([NO_3^-] = 0.05 mol L^{-1}) in dark and (D) under irradiation, (E) GA/NO_3^- ([NO_3^-] = 0.5 mol L^{-1}) in dark, and (F) under irradiation.

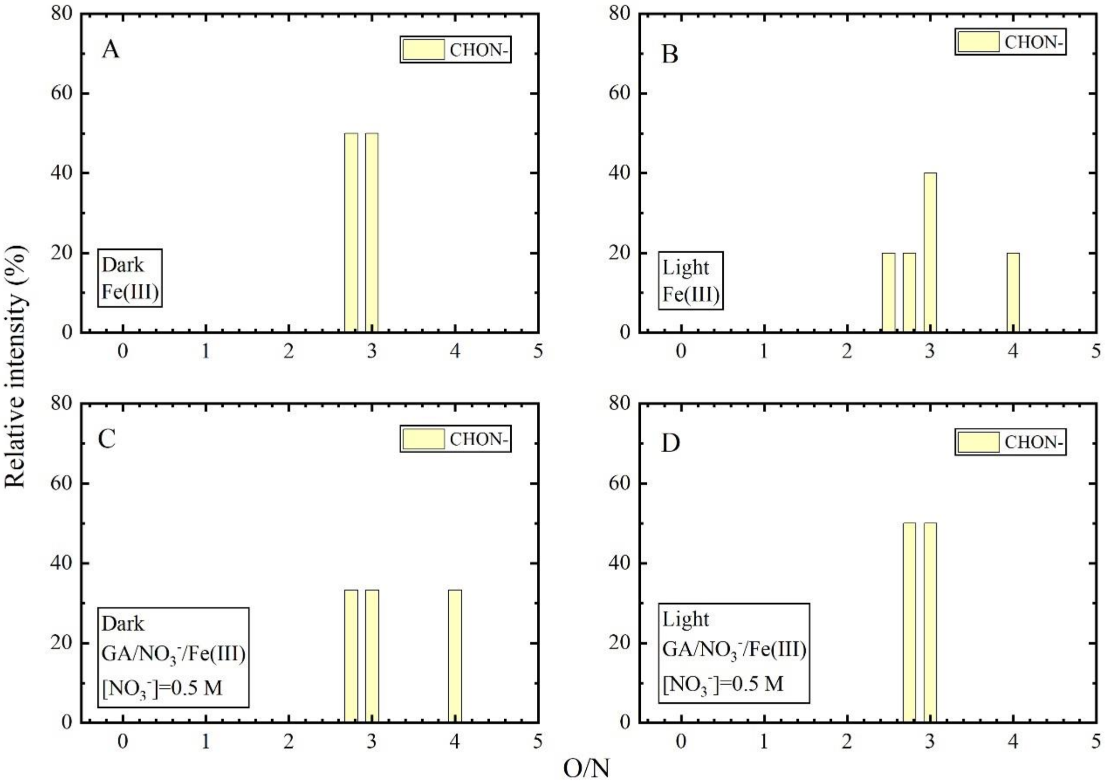

Figure S4: N-containing organic compounds are divided into subgroups related to the O/N ratio in their chemical composition. The y-axis represents the contribution of each subgroup to the total intensity of CHON compounds in ESI^- ion mode detected in heterogeneous reaction of NO_2 with GA/Fe(III) (A) in dark, and (B) under irradiation, (C) GA/NO_3^-/Fe(III) ([NO_3^-] = 0.5 mol L^{-1}) in dark, and (D) under irradiation.

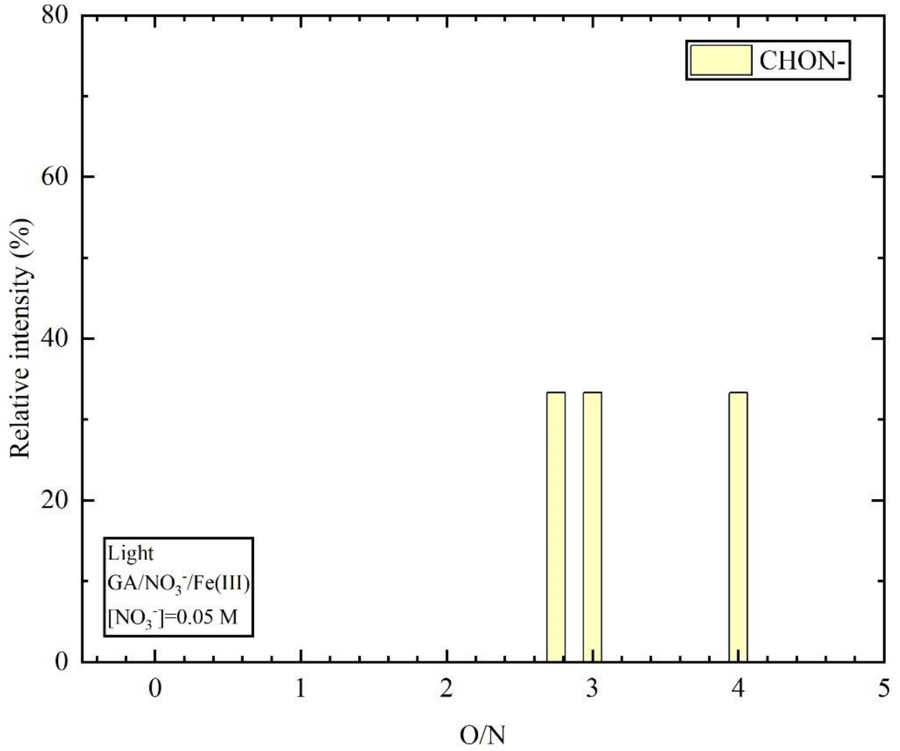

Figure S5: N-containing organic compounds are divided into subgroups related to the O/N ratio in their chemical composition. The y-axis represents the contribution of each subgroup to the total intensity of CHON compounds in ESI^- ion mode detected in heterogeneous reaction of NO_2 with GA/NO_3^-/Fe(III) ([NO_3^-] = 0.05 mol L^{-1}) under irradiation.

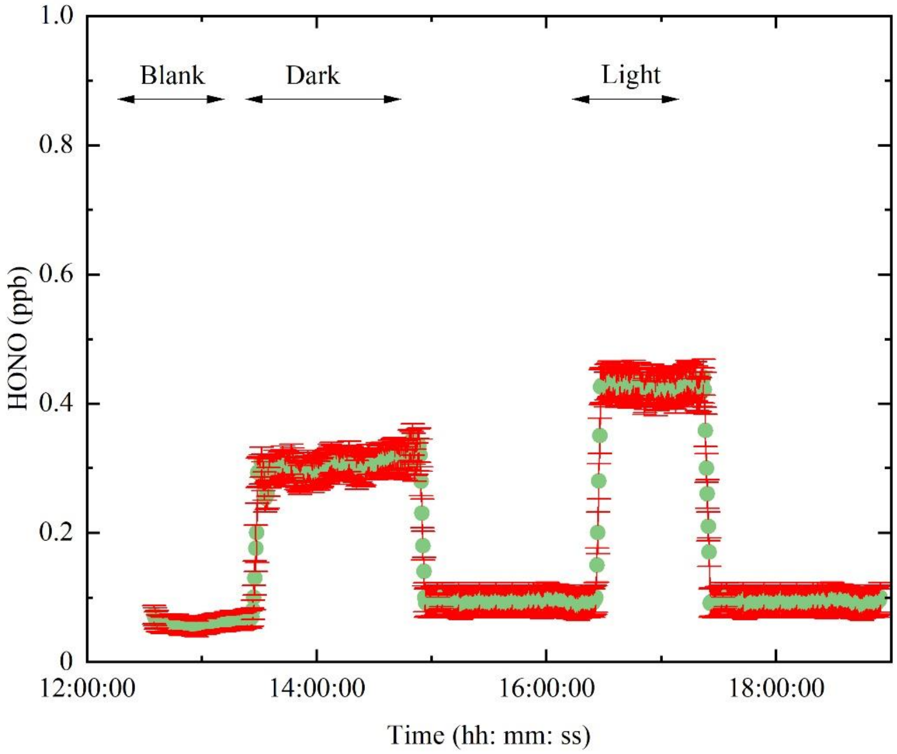

Figure S6: Typical signal of HONO formed during the reaction of NO_2 (50 ppb) with GA (1×10^{-4} mol L^{-1}) in the presence of NO_3^- ($[NO_3^-]$ = 0.05 mol L^{-1}) in dark and under irradiation. The error bar on HONO signal (red) corresponds to 10% uncertainties in the measurements of HONO by LOPAP.

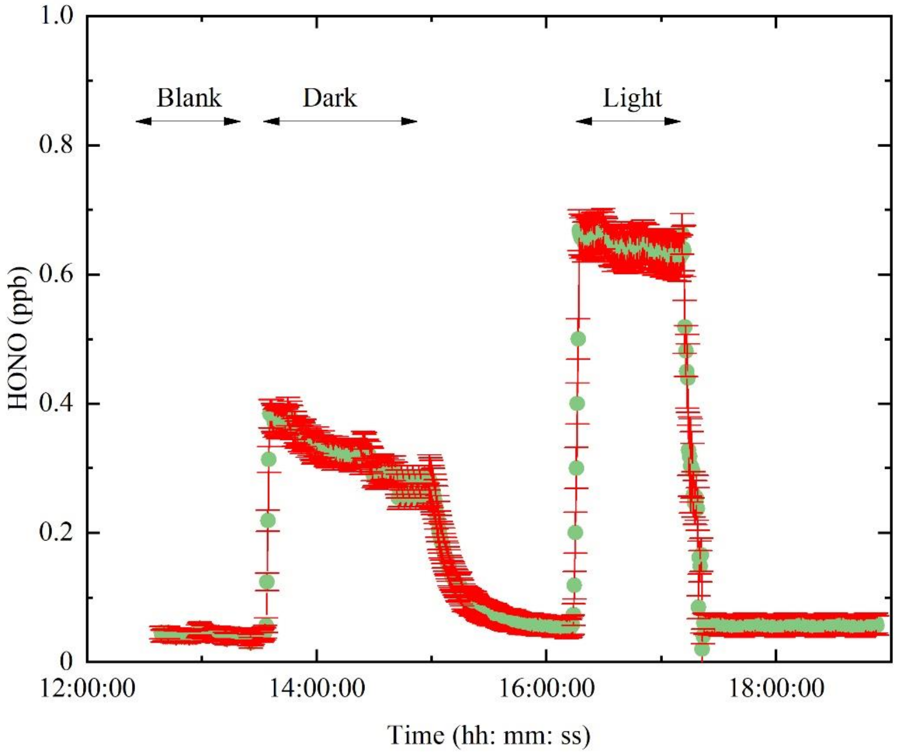

Figure S7: Typical signal of HONO (green) formed during the reaction of NO_2 (50 ppb) with GA (1×10^{-4} mol L^{-1}) with GA in the presence of Fe(III) (3×10^{-5} mol L^{-1}), in dark and under irradiation. The error bar on HONO signal (red) corresponds to 10% uncertainties in the measurements of HONO by LOPAP.

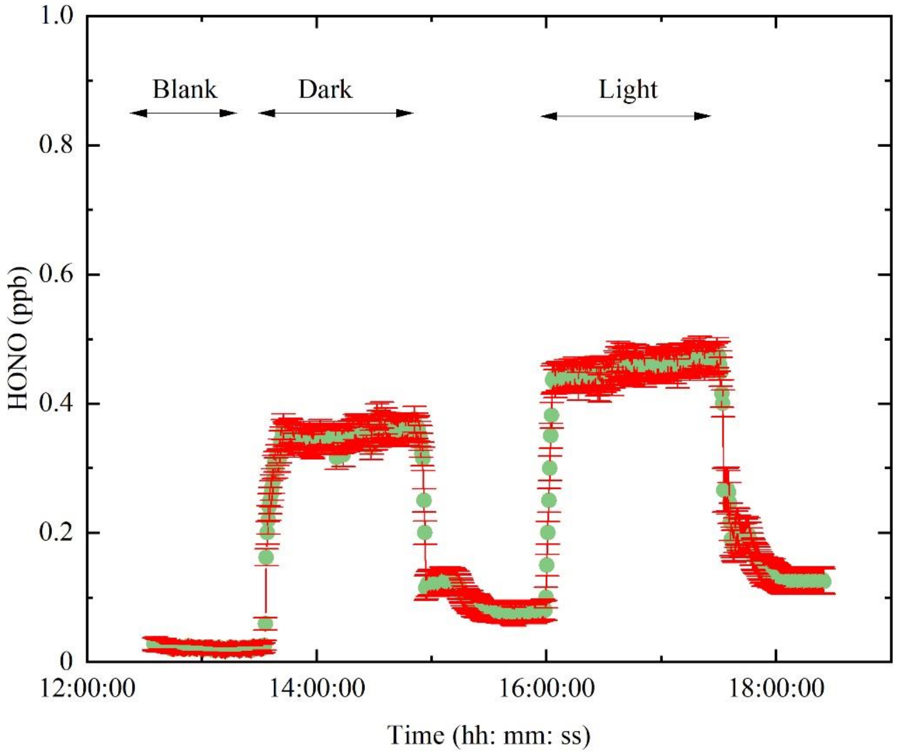

Figure S8: Typical signal of HONO (green) formed during the reaction of NO_2 (50 ppb) with GA (1×10^{-4} mol L^{-1}) in the presence of NO_3^- (0.05 mol L^{-1})/Fe^{3+} (1×10^{-6} mol L^{-1}) in dark and under irradiation. The error bar on HONO signal (red) corresponds to 10% uncertainties in the measurements of HONO by LOPAP.

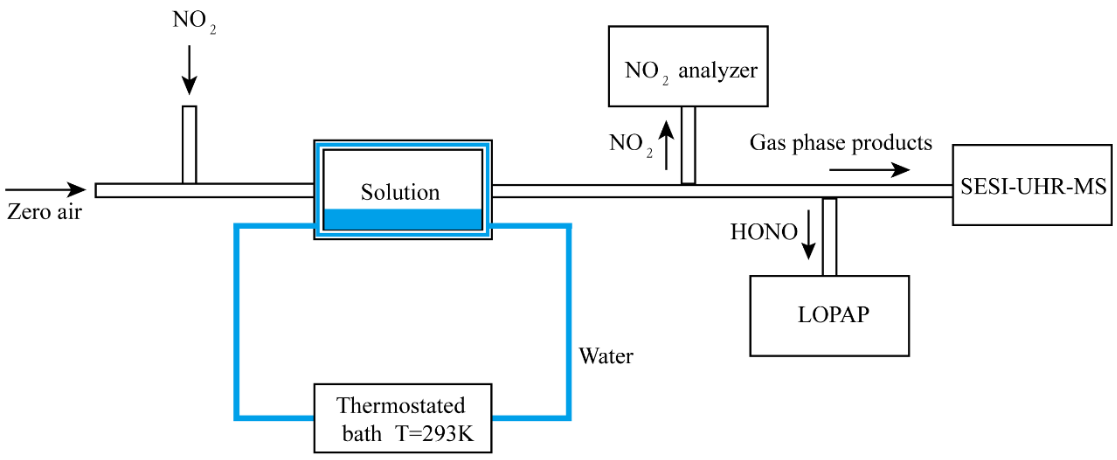

Figure S9: Simplified illustration of the experimental set up used in this study.

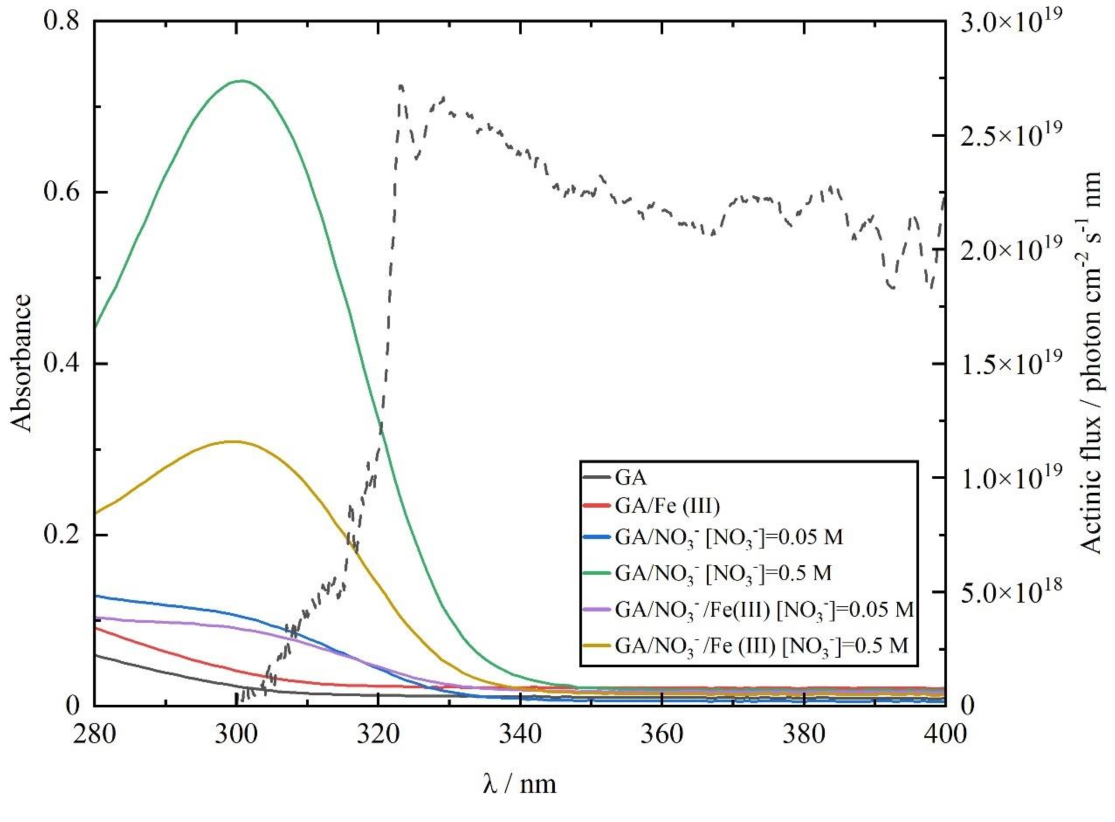

Figure S10: The absorption spectra of GA (1×10^{-4} mol L^{-1}) in the presence of $NaNO_3$ (I_{eff} = 0.05 M, 0.5 M)/Fe(III) (1×10^{-6} mol L^{-1}), the photon flux of Xe lamp (black dashed line). The left axis corresponds to the absorption spectra of GA in presence of $NaNO_3$ and Fe(III), and the right axis corresponds to the spectral irradiance of xenon lamp.

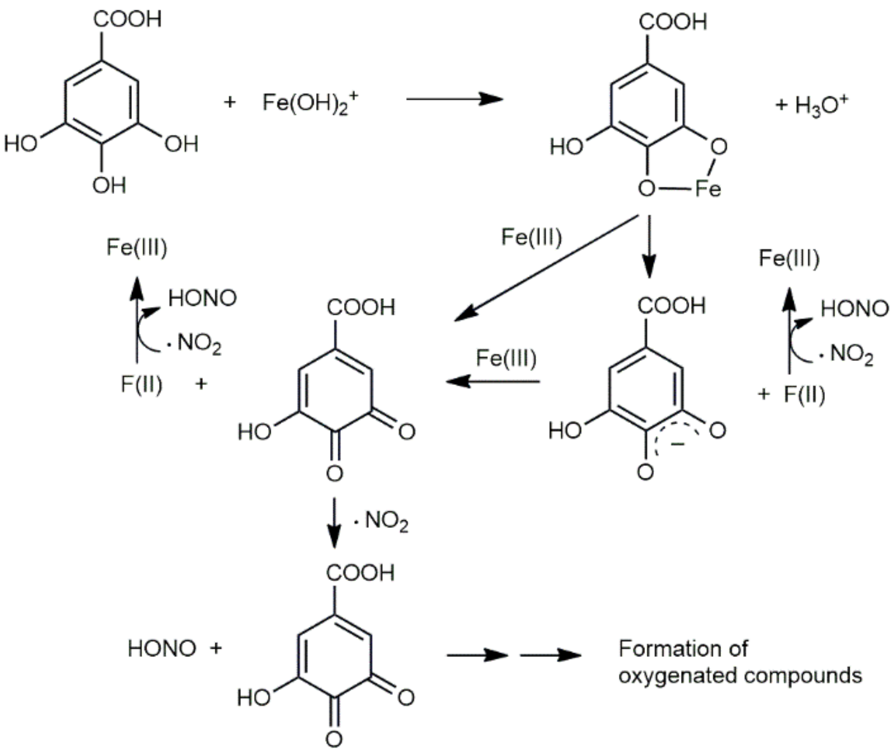

Figure S11: The tentative pathway of HONO formation in dark.

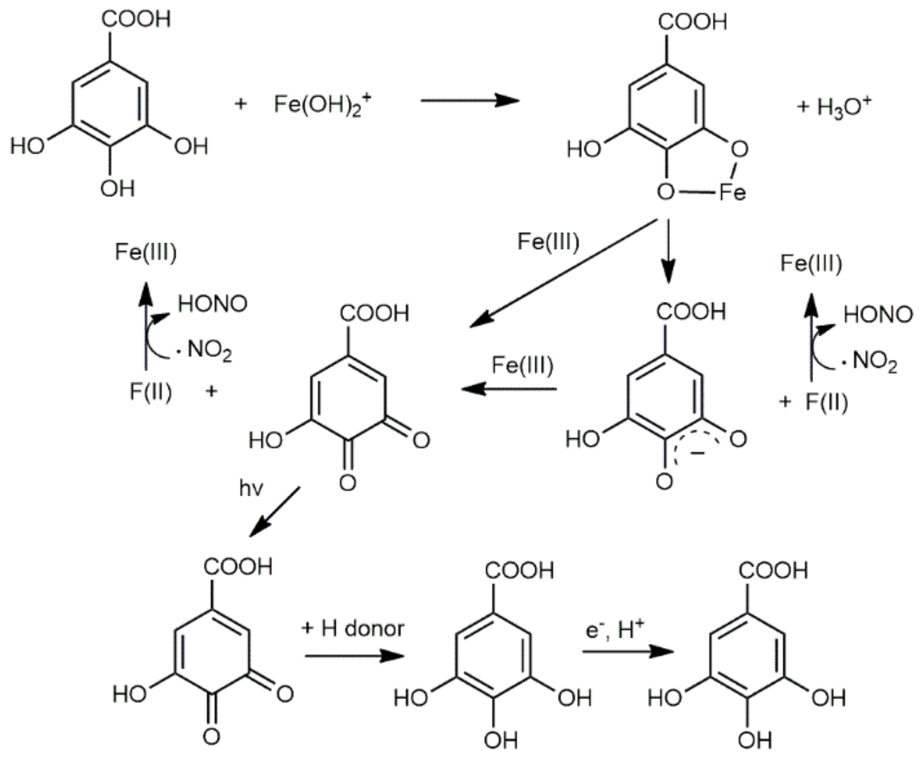

Figure S12: The tentative pathway of HONO formation under irradiation.

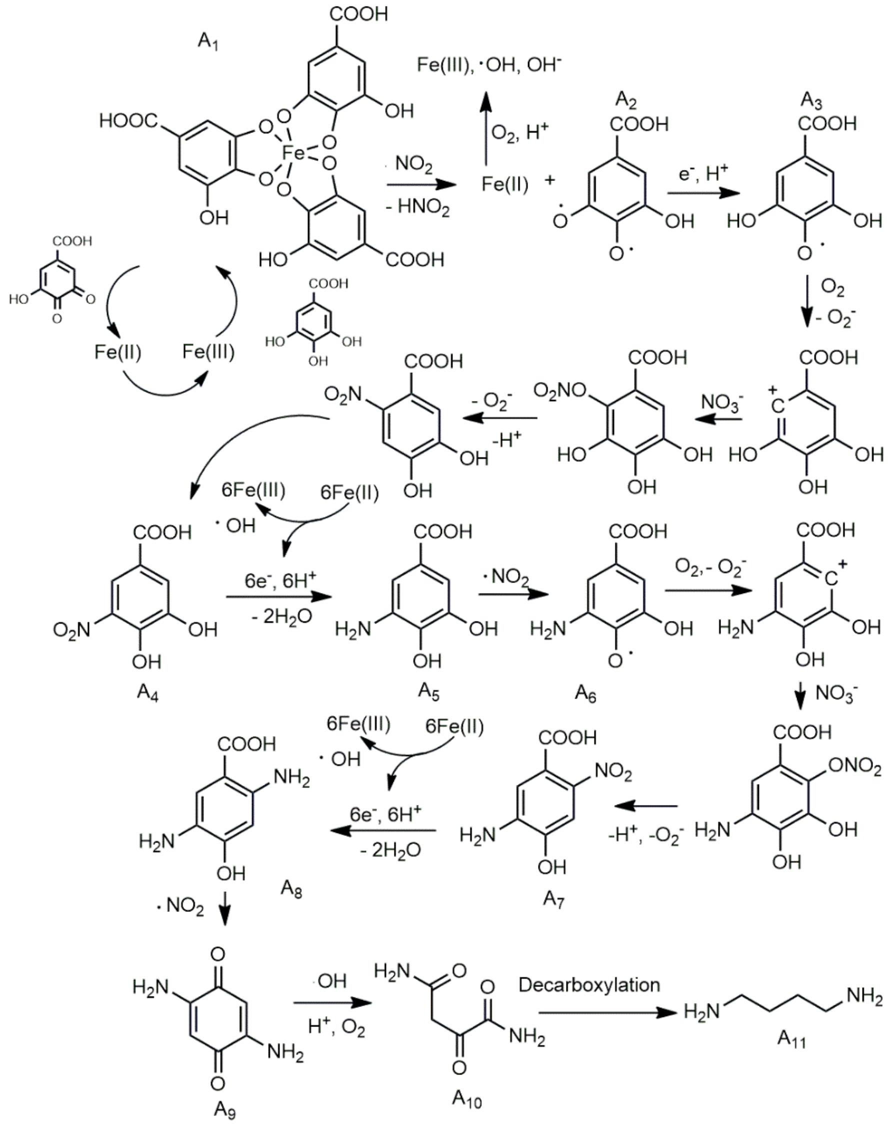

Figure S13: A tentative reaction mechanism describing the formation of putrescine (A_{11}) in the presence of Fe(III) and NO_3^- in dark.

Table S1: Observed compounds formed upon heterogeneous NO_2 processing of GA/NO_3^-/Fe(III) [NO_3^-] = 0.05 mol L^{-1} in dark. Formulae are shown in their neutral forms.

NO.	Mode	m/z (exp)	Formula	Tentative structures	DBE	Reference
1	ESI+	103.0388	$C_4H_6O_3$	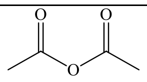	2	[1,2]

2	ESI+	117.0544	$C_5H_8O_3$	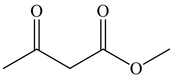	2	1,2
3	ESI+	121.0646	C_8H_8O	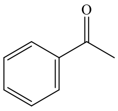	5	1
4	ESI-	91.99809	CH_3O_4N	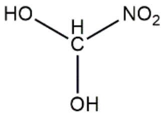	1	
5	ESI+	131.0703	$C_6H_{10}O_3$	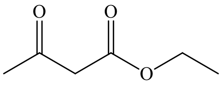	2	1,2
6	ESI+	137.0596	$C_8H_8O_2$	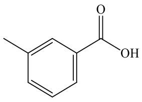	5	1,2
7	ESI+	139.0389	$C_7H_6O_3$	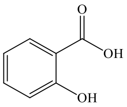	5	1
8	ESI+	139.1117	$C_9H_{14}O$	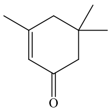	3	1
9	ESI+	141.0909	$C_8H_{12}O_2$	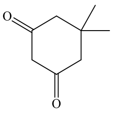	3	1,2
10	ESI+	143.1065	$C_8H_{14}O_2$	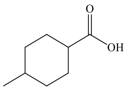	2	1,2
11	ESI+	149.0232	$C_8H_4O_3$	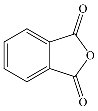	7	1
12	ESI+	163.0388	$C_9H_6O_3$	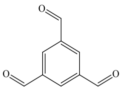	7	1
13	ESI+	163.1327	$C_8H_{18}O_3$	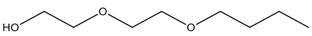	0	1
14	ESI+	183.0125	$C_4H_6O_8$	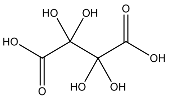	2	
15	ESI+	191.164	$C_{10}H_{22}O_3$	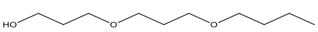	0	1
16	ESI+	195.0651	$C_{10}H_{10}O_4$	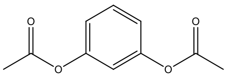	6	1
17	ESI+	199.1691	$C_{12}H_{22}O_2$	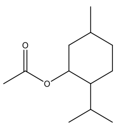	2	1
18	ESI+	203.1276	$C_{10}H_{18}O_4$	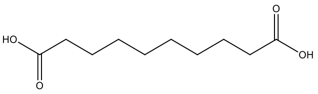	2	1

19	ESI+	207.1589	$C_{10}H_{22}O_4$	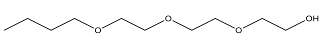	0	1
20	ESI+	217.1797	$C_{12}H_{24}HO_3$	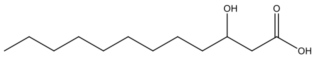	1	
21	ESI+	279.159	$C_{16}H_{22}O_4$	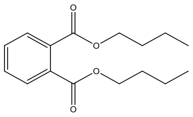	6	1
22	ESI+	287.2216	$C_{16}H_{30}O_4$	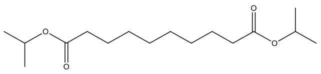	2	1
23	ESI+	114.0912	$C_6H_{11}ON$	/	2	1-3
24	ESI+	128.1068	$C_7H_{13}ON$	/	2	2
25	ESI+	130.1227	$C_7H_{15}ON$	/	1	
26	ESI+	144.1382	$C_8H_{17}ON$	/	1	
27	ESI+	150.0912	$C_9H_{11}ON$	/	5	2
28	ESI+	158.1538	$C_9H_{19}ON$	/	1	2
29	ESI+	172.1694	$C_{10}H_{21}ON$	/	1	
30	ESI+	182.0091	$C_7H_3O_5N$	/	7	
31	ESI+	184.0756	$C_{12}H_9ON$	/	9	
31	ESI+	188.1068	$C_{12}H_{13}ON$	/	7	
33	ESI+	204.1018	$C_{12}H_{13}O_2N$	/	7	
34	ESI+	218.1174	$C_{13}H_{15}O_2N$	/	7	
35	ESI+	232.1331	$C_{14}H_{17}O_2N$	/	7	
36	ESI+	240.232	$C_{15}H_{29}ON$	/	2	
37	ESI+	131.1179	$C_6H_{14}ON_2$	/	1	2
38	ESI+	219.1127	$C_{12}H_{14}O_2N_2$	/	7	
39	ESI+	261.1596	$C_{15}H_{20}O_2N_2$	/	7	
40	ESI+	164.0422	$C_2H_5O_4N_5$	/	3	
41	ESI+	175.131	$C_5H_{14}ON_6$	/	2	
42	ESI+	196.0684	$C_4H_5ON_9$	/	7	
43	ESI+	280.1623	$C_{10}H_{17}ON_9$	/	7	
44	ESI+	170.0963	$C_{12}H_{11}N$	/	8	1
45	ESI+	174.1276	$C_{12}H_{15}N$	/	6	
46	ESI+	176.1433	$C_{12}H_{17}N$	/	5	1
47	ESI+	188.1432	$C_{13}H_{17}N$	/	6	1
48	ESI-	246.9785	$CH_4O_{11}N_4$	/	2	
49	ESI-	196.9823	$C_6H_2O_6N_2$	/	7	
50	ESI-	228.988	$C_{10}H_2O_5N_2$	/	11	
51	ESI+	115.0946	NA	/		
52	ESI+	135.8566	NA	/		
53	ESI+	136.186	NA	/		

54	ESI+	184.0049	NA	/

Table S2: Observed compounds formed upon heterogeneous NO_2 processing of GA/NO_3^-/Fe(III) [NO_3^-] = 0.05 mol L^{-1} under simulated sunlight irradiation. Formulae are shown in their neutral forms.

NO.	Mode	m/z (exp)	Formula	Tentative structures	DBE	Reference
1	ESI+	75.04391	$C_3H_6O_2$	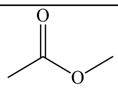	1	1
2	ESI+	85.06461	C_5H_8O	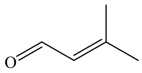	2	1,2
3	ESI+	101.0595	$C_5H_8O_2$	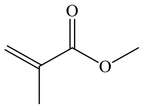	2	1,2
4	ESI+	103.0388	$C_4H_6O_3$	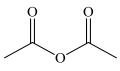	2	1,2
5	ESI+	109.101	C_8H_{12}	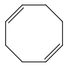	3	
6	ESI+	117.0545	$C_5H_8O_3$	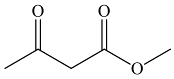	2	1,2
7	ESI+	121.0646	C_8H_8O	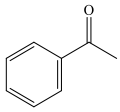	5	1
8	ESI+	123.1167	C_9H_{14}	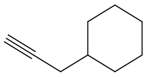	3	
9	ESI+	125.1323	C_9H_{16}	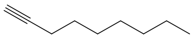	2	
10	ESI+	127.1116	$C_8H_{14}O$	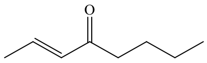	2	1,2
11	ESI+	141.1273	$C_9H_{16}O$	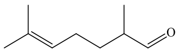	2	1
12	ESI+	143.143	$C_9H_{18}O$	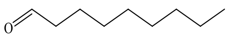	1	1

Table S3: Observed compounds upon heterogeneous NO_2 processing of GA in dark. Formulae are shown in their neutral forms.

NO.	Mode	*m/z* (exp)	Formula	Tentative structures	DBE
1	ESI-	184.9825	$C_5H_2O_6N_2$	/	6
2	ESI-	196.9823	$C_6H_2O_6N_2$	/	7
3	ESI-	265.9793	$C_7HO_7N_5$	/	10

Table S4: Observed compounds upon heterogeneous NO_2 processing of GA under simulated sunlight irradiation. Formulae are shown in their neutral forms.

NO.	Mode	*m/z* (exp)	Formula	Tentative structures	DBE
1	ESI-	131.0701	$C_6H_{12}O_3$	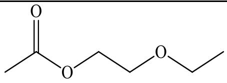	1
2	ESI-	197.0952	$C_{14}H_{14}O$	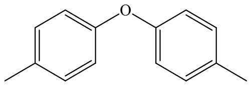	8
3	ESI-	138.0183	$C_6H_5O_3N$	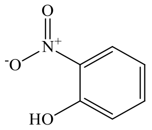	5
4	ESI-	196.9823	$C_6H_2O_6N_2$	/	7

Table S5: Observed compounds upon heterogeneous NO_2 processing of GA/Fe(III) in dark. Formulae are shown in their neutral forms.

NO.	Mode	*m/z* (exp)	Formula	Tentative structures	DBE
1	ESI-	196.9824	$C_6H_2O_6N_2$	/	7

Table S6: Observed compounds upon heterogeneous NO_2 processing of GA/Fe(III) under simulated sunlight irradiation. Formulae are shown in their neutral forms.

NO.	Mode	m/z (exp)	Formula	Tentative structures	DBE
1	ESI-	138.0184	$C_6H_5O_3N$	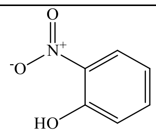	5
2	ESI-	196.9824	$C_6H_2O_6N_2$	/	7
3	ESI-	228.9883	$C_{10}H_2O_5N_2$	/	11

Table S7: Observed compounds upon heterogeneous NO_2 processing of GA/NO_3^- [NO_3^-] = 0.05 mol L^{-1} in dark. Formulae are shown in their neutral forms.

NO.	Mode	m/z (exp)	Formula	Tentative structures	DBE
1	ESI-	133.0242	$C_3H_6O_4N_2$	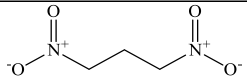	2
2	ESI-	138.0183	$C_6H_5O_3N$	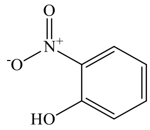	5
3	ESI-	147.0397	$C_9H_8O_2$	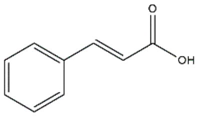	6
4	ESI-	161.0552	$C_{10}H_{10}O_2$	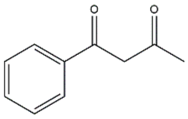	6

Table S8: Observed compounds upon heterogeneous NO_2 processing of GA/NO_3^- [NO_3^-] = 0.05 mol L^{-1} under simulated sunlight irradiation. Formulae are shown in their neutral forms.

NO.	Mode	m/z (exp)	Formula	Tentative structures	DBE	Reference.
1	ESI+	101.0595	$C_5H_8O_2$	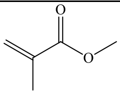	2	[1,2]
2	ESI+	121.0646	C_8H_8O	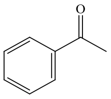	5	[1]
3	ESI+	127.1116	$C_8H_{14}O$	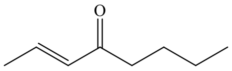	2	[1]
4	ESI+	141.1273	$C_9H_{16}O$	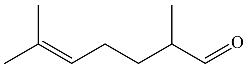	2	[1]
5	ESI+	177.1636	$C_{13}H_{20}$	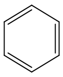	4	[1]
6	ESI+	195.1743	$C_{13}H_{22}O$	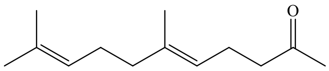	3	[1,2]

Table S9: Observed compounds upon heterogeneous NO_2 processing of GA/NO_3^- [NO_3^-] = 0.5 mol L^{-1} in dark. Formulae are shown in their neutral forms.

NO.	Mode	m/z (exp)	Formula	Tentative structures	DBE
1	ESI-	196.9823	$C_6H_2O_6N_2$	/	7

Table S10: Observed compounds formed upon heterogeneous NO_2 processing of GA/NO_3^- [NO_3^-] = 0.5 mol L^{-1} under simulated sunlight irradiation. Formulae are shown in their neutral forms.

NO.	Mode	m/z (exp)	Formula	Tentative structures	DBE
1	ESI-	91.99809	CH_3O_4N	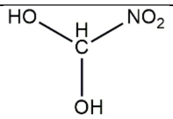	1
2	ESI-	265.9793	$C_7HO_7N_5$	/	10

Table S11: Observed compounds formed upon heterogeneous NO_2 processing of GA/Fe(III)/NO_3^-/Fe(III) [NO_3^-] = 0.5 mol L^{-1} in dark. Formulae are shown in their neutral forms.

NO.	Mode	m/z (exp)	Formula	Tentative structures	DBE	Reference
1	ESI+	75.04391	$C_3H_6O_2$	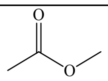	1	[1]
2	ESI+	85.06461	C_5H_8O	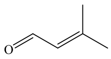	2	[1,2]
3	ESI+	87.08028	$C_5H_{10}O$	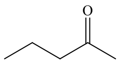	1	[1]
4	ESI+	103.0388	$C_4H_6O_3$	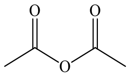	2	[1,2]
5	ESI+	117.0545	$C_5H_8O_3$	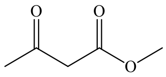	2	[1,2]
6	ESI+	121.0646	C_8H_8O	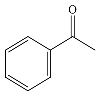	5	[1]
7	ESI+	129.0909	$C_7H_{12}O_2$	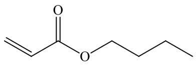	2	[1,2]
8	ESI+	131.0703	$C_6H_{10}O_3$	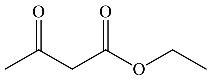	2	[1,2]

Table S12: Observed compounds formed upon heterogeneous NO_2 processing of GA/Fe(III)/NO_3^-Fe(III) [NO_3^-] = 0.5 mol L^{-1} under simulated sunlight irradiation. Formulae are shown in their neutral forms.

NO.	Mode	m/z (exp)	Formula	Tentative structures	DBE	Reference
1	ESI+	85.06461	C_5H_8O	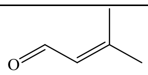	2	[1,2]
2	ESI+	101.0595	$C_5H_8O_2$	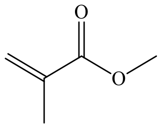	2	[1,2]
3	ESI+	103.0388	$C_4H_6O_3$	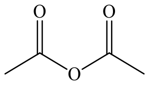	2	[1,2]
4	ESI+	109.101	C_8H_{12}	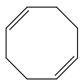	3	
5	ESI+	117.0545	$C_5H_8O_3$	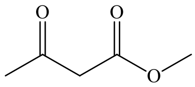	2	[1,2]
6	ESI+	121.0646	C_8H_8O	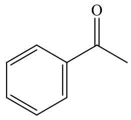	5	[1]
7	ESI+	123.1167	C_9H_{14}	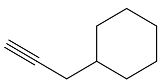	3	
8	ESI+	127.1116	$C_8H_{14}O$	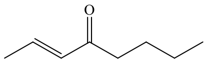	2	[1,2]
9	ESI+	131.0703	$C_6H_{10}O_3$	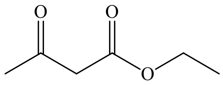	2	[1,2]
10	ESI+	141.1273	$C_9H_{16}O$	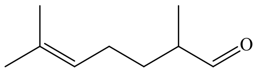	2	[1]
11	ESI+	143.1429	$C_9H_{18}O$	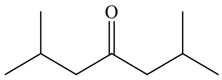	1	[1]

Table S13: The variation of pH during the heterogeneous NO_2 processing with GA in dark and under irradiation.

GA (mol L^{-1})	$NaNO_3$(mol L^{-1})	$FeCl_3$•$6H_2O$ (mol L^{-1})	Initial pH	pH after reaction	
				Dark	Light
10^{-4}			5	11.71	10.74
$10^{-}4$	0.05		5	11.44	10.47
10^{-4}	0.5		5	11.56	10.59
10^{-4}		10^{-6}	5	11.8	11.36
10^{-4}	0.05	10^{-6}	5	11.7	10.60
10^{-4}	0.5	10^{-6}	5	11.5	10.12

Supplementary References

1 Wang, X. *et al.* Chemical Characteristics of Organic Aerosols in Shanghai: A Study by Ultrahigh-Performance Liquid Chromatography Coupled With Orbitrap Mass Spectrometry. *J. Geophys. Res.: Atmos.* **122**, 11703-11722 (2017).

2 Wang, Y. *et al.* Interfacial Ozone Oxidation Chemistry at a Riverine Surface Microlayer as a Source of Nitrogen Organic Compounds. *Environ. Sci. Technol. Lett.* **9**, 493-500 (2022).

3 Laskin, A., Smith, J. S. & Laskin, J. Molecular Characterization of Nitrogen-Containing Organic Compounds in Biomass Burning Aerosols Using High-Resolution Mass Spectrometry. *Environ. Sci. Technol.* **43**, 3764-3771 (2009).
