# Supplementary material for: Formation of nitrogen-containing gas phase products from the heterogeneous (photo)reaction of NO2 with gallic acid
Source: Commun Chem. 2023 Sep 16;6:198. doi: 10.1038/s42004-023-01003-3 (PMC10505156; doi:10.1038/s42004-023-01003-3)
Supplement: Supplementary file 3 — Description of Additional Supplementary Files [file 42004_2023_1003_MOESM3_ESM.pdf]

Description of Additional Supplementary Files

File name: Supplementary Data 1

Description: Source data for graphs and charts
